# Antibiotic use in South Korea from 2007 to 2014: A health insurance database-generated time series analysis

**DOI:** 10.1371/journal.pone.0177435

**Published:** 2017-05-17

**Authors:** Juhee Park, Euna Han, Soo Ok Lee, Dong-Sook Kim

**Affiliations:** 1 Department of Research, Health Insurance Review & Assessment Service, Wonju, Korea; 2 College of Pharmacy, Yonsei Institute of Pharmaceutical Science, Yonsei University, Seoul, Korea; Kaohsiung Medical University, TAIWAN

## Abstract

**Background:**

Inappropriate antibiotic use significantly contributes to antibiotic-resistance, resulting in reduced antibiotic efficacy and increasing physical burden and cost of disease. The goal of this study was to explore antibiotic usage patterns in South Korea using 2007–2014 health insurance claims data.

**Methods:**

We used the Health Insurance Review & Assessment Service data, which represents nearly the entire population of South Korea, to discern patterns in antibiotic prescribing practices. The daily dose, as defined by the World Health Organization ([defined daily doses]/1000 inhabitants/day, [DID]), was used as a measure of antibiotic use. Subgroup analyses were performed on the basis of patient characteristics (sex, age, and disease) and provider characteristics (type of medical institution).

**Results:**

Antibiotic use in DID increased from 23.5 in 2007 to 27.7 in 2014. The ≤ 6 years old age group showed the highest level of usage at 59.21 DID in 2014, and showed an increasing trend each year. DIDs of beta-lactam antibacterials, penicillins (J01C), other beta-lactam antibacterials (J01D), lincosamides and streptogramins (J01F), quinolone antibacterials (J01M), and other antibacterials (J01X) increased over time.

**Conclusion:**

This study provides valuable statistics regarding antibiotic usage in South Korea; this is important for guiding health policy with regard to antibiotic usage. There is a need for further study exploring antibiotics use and resistance.

## Introduction

Antibiotics are pivotal in the treatment of infectious diseases. However, the inappropriate use and overuse of antibiotics are emerging as major global issues because of the increased number of resistant bacteria, reduced antibiotic efficacy, multiple infections, and increased medical expenses [1]. The development of new antibiotics helps in the fight against resistant bacteria, but resistance to new antibiotics is emerging more rapidly than expected, and antibiotic development is becoming increasingly difficult [2, 3]. An increase in antibiotic-resistant bacteria has a considerable impact on disease treatment and increases the economic burden of infection. The annual cost associated with antibiotic-resistant infections in the US is estimated to be between $21 billion to $34 billion (US dollars) [3].

In South Korea, antibiotic use has shown a decreasing trend since separating the prescribing and dispensing of drugs in 2000. However, the level of antibiotic usage in South Korea (28 defined daily doses [DDD] per 1,000 habitants per day [DID]) is still much higher than the average for other Organisation for Economic Co-operation and Development (OECD) countries (18 DID) as of 2012 [4].

To resolve this issue, since 2001, South Korea has implemented the Project to Assess the Appropriateness of Drug Provision, evaluating the antibiotic prescription rate for upper respiratory tract infections per each prescriber to assess prescribing habits [5]. Specifically, the Health Insurance Review & Assessment Service (HIRA) calculates antibiotic prescription rates for acute nasopharyngitis (common cold), acute sinusitis, acute pharyngitis, acute tonsillitis, acute laryngitis and tracheitis, acute obstructive laryngitis, and epiglottitis (International Classification of Disease [ICD]-10 codes, J00-J06), and provides medical institutions with these data [5]. The information is also shared with the public via the HIRA website. The outpatient antibiotic prescription rate decreased from 42.39% in 2002 to 22.57% in 2014, and the antibiotic prescription rate for upper respiratory tract infection decreased from 73.33% in 2002 to 43.37% in 2014. However, problems of overuse still exist, particularly in outpatient clinics as evidenced by an antibiotic prescription rate of 25.8% compared to a rate of 5.11% in tertiary hospitals in 2014 [5].

The increase in antibiotic-resistant bacteria has been a major public health concern in South Korea. Multidrug-resistant *Streptococcus pneumoniae*, community-associated methicillin-resistant *Staphylococcus aureus*, and extended-spectrum β-lactamase producing *Enterobacteriaceae* are major issues in antibiotic resistance [6]. Seventy-three percent of *S*. *aureus* infections were methicillin-resistant in a survey of hospital samples in 2012 [2]. In Asian countries, including South Korea, over 50% of *S*. *pneumoniae* showed penicillin resistance [7], and 80.6% showed macrolide resistance in South Korea [8]. The appropriate use of antibiotics has been emphasized as key for controlling antibiotic resistance [9]. The World Health Organization (WHO) defines appropriate antibiotic use as cost-effective usage, maximizing the therapeutic effect while minimizing toxicity and resistance [2]. Unfortunately, the correlation between the level of antibiotic use and the development of resistance has been shown in a large number of studies [10, 11].

The aim of the present study was to investigate the epidemiological characteristics of antibiotic use at a national level in South Korea. The investigation of antibiotic usage patterns is important for detecting the inappropriate use of antibiotics and for designing public health intervention strategies to promote appropriate use. Although previous studies have investigating the use of antibiotics with claims data [12, 13], the majority used either sample data that was not representative of the entire population or were limited to a single year, making it difficult to examine temporal changes. No studies have used claims data, representing the entire national population, to examine antibiotic use patterns outside of South Korea. The goal of this study was to explore antibiotic consumption in South Korea using 2007–2014 health insurance claims data. This study presents data from an Asian country where, similar to many countries, appropriate antibiotic use has been a major public health concern.

## Methods

### Data

This analysis used the National Health Insurance claims database (HIRA claims data) from 2007–2014. This database is representative of the entire population of approximately 50 million South Koreans and includes compulsory beneficiaries of National Health Insurance or the Medical Aid for low-income members of the population. The HIRA claims data contain each patient’s unique encrypted identification number, age, sex, primary diagnosis, secondary diagnosis, surgical or medical treatment administered, whether the individual was an inpatient or outpatient, type of insurance (National Health Insurance or Medical Aid), medical expenses, medical institution identification number, and prescriptions. The diagnoses were coded according to the International Classification of Disease, Tenth Revision (ICD-10). The drug names were coded according to the Korean national drug code system; almost all antibiotics are listed as reimbursable drugs under the National Health Insurance. Therefore, our data represent antibiotic use for nearly the entire South Korean population for the given period.

Medical institutions included in this study were tertiary hospitals, secondary hospitals, hospitals, clinics, dental hospitals, dental clinics, and public health institutions. We analyzed claims for health care services submitted to HIRA by these medical institutions for patients treated between January 01, 2007 and December 31, 2014.

### Definition of antibiotics

Antibiotics were defined as drugs classified as J01 (antibacterials for systemic use) according to WHO’s Anatomic Therapeutic Chemical (ATC) classification [12]. To measure the number of drugs, we recoded the Korean national drug code according to the WHO-ATC classification system. The unit of the administered drug was applied as the chemical subgroup level (ATC-4, 5 digits of ATC) [12]. In our analysis, we included antibiotics that were listed from 2007 to 2014.

### Outcome measures

Drug use was assessed using drug consumption in units of DDD, which allows standard consumption to be calculated by adjusting variations in doses and units for each antibiotic product [12]. DDD enables a comparative analysis of consumption trends over time within a country and across different countries.

Annual usage was calculated as DDD/1,000 inhabitants/day (hereafter DID) as shown in Eq 1 below. Based on the WHO daily maintenance dose for an adult weighing 70 kg, DDD was calculated for each individual formulation. To calculate drug usage and costs for different demographic characteristics, we used yearly population projection data from South Korean statistics for each year from 2007 to 2014.

Drugconsumption(DDDper1,000inhabitantsperday)=(Amountofdrugconsumedin1year(mg))÷(DDD(mg)×365days×totalpopulation)×1,000persons (1)

### Data analysis

We analyzed antibiotic usage for the entire South Korean population and for various subgroups including groups based on patient demographics, utilization type, and prescribing practice setting. Patient age was defined as age on the date of the encounter and was grouped into four categories: ≤6 y, 7–19 y, 20–64 y, and date of the encounter and was grouped into four categories: ≤6 y, 7ups including groups based on patient demographics, utilization type, and fourth ATC classification level.

We used an augmented Dickey-Fuller test for the null hypothesis of unit root process for each pharmacological subgroup as in Eq (2).

$${\Delta y}_{t}=(1-\rho )\beta_{0}+{(\rho -1)y}_{y-1}+\epsilon_{t}$$ (2)

where *t* represents the time trend, and *β*, *δ*, and *ζ* are parameters to estimate, and *ε*_*t*_ is identically independently distributed stochastic error. Rejection of the null hypothesis H0: *ρ* = 1 in Eq (2) implies a weakly dependent process in the *y*, whereas failure to reject implies a potential unit root process in the y.

We used a structural autoregressive moving average (ARMA) model with the autoregressive process of order one (AR(1)) and moving average process of order one (MA(1)) as in Eq (3). The first differencing was used as in Eq (4) only when a highly persistent time series was found in the ARMA model, i.e., when we failed to reject the null hypothesis in the Dickey-Fuller test. A separate model was estimated by eight pharmacological subgroups based on the fourth ATC classification level (ATC-3).

$$y_{t}=(1-\rho_{1})\beta_{0}+\rho_{1}y_{t-1}+\theta_{1}\epsilon_{t-1}+\epsilon_{t}$$ (3)

$${\Delta y}_{t}=(1-\rho_{1})\beta_{0}+\rho_{1}\Delta y_{t-1}+\theta_{1}\varepsilon_{t-1}+\varepsilon_{t}$$ (4)

where y_t_ is DID at time t, β is the size of the change in y_t_, ρ is the first-order autocorrelation parameter, *θ* is the first-order moving average parameter, and *ε*_*t*_ and *ϵ*_*t*_ are identically independently distributed white noise component of the ARMA model at time t.

SAS 9.1 (SAS, Cary, NC, USA) STATA SE 14.0 (Stata Corporation, College Station, TX, USA) were used for all analyses.

### Ethic statement

The study design was approved by the Research Ethics Committees which is predecessor of Institutional Review Board of the HIRA.

## Results

### Antibiotic use patterns by subgroup

Total antibiotic usage increased by 4.2 DID from 2007 (23.5 DID) to 2014 (27.7 DID). For inpatient services, antibiotic usage increased from 2.4 DID in 2007 to 2.8 DID in 2012, but decreased to 2.6 DID in 2014. Outpatient usage fluctuated in a manner similar to total usage, although there was a downward trend in 2011 when various health policies were implemented. Age groups were categorized as ≤6 years old, 7–19 years old, 20–64 years old, and ≥65 years old. The ≤6 years old age group showed the highest level of usage at 59.21 DID in 2014 and showed an increasing trend each year and a particularly large increase in 2010 (Table 1).

**Table 1:** Total antibiotic consumption for the entire study population and by sex, age, and service type subgroups (daily defined dose/1,000 inhabitants/day).

Year	2007	2008	2009	2010	2011	2012	2013	2014
**Total care**	23.5	24.8	25.8	26.7	26.0	26.8	26.7	27.7
**(Percentage increase)**	-	(6.28%)	(4.55%)	(3.88%)	(-1.69%)	(3.50%)	(0.03%)	(4.13%)
**By service type**								
**Inpatient care**	2.4	2.5	2.5	2.6	2.7	2.8	2.7	2.6
**Outpatient care**	21.0	22.3	23.3	24.0	23.3	24.0	24.0	25.0
**By sex**								
**Male**	22.22	23.36	24.19	25.05	24.52	25.27	25.25	26.10
**Female**	24.75	26.20	27.38	28.27	27.51	28.34	28.14	29.27
**By age group**								
**≤6 years old**	47.00	49.84	51.95	59.37	58.72	60.77	59.69	59.21
**07–19 years old**	17.51	18.89	21.99	22.16	20.78	20.89	21.02	23.15
**20–64 years old**	20.96	22.00	22.46	22.92	22.48	23.06	22.95	23.80
**≥65 years old**	33.95	35.89	36.07	36.49	35.45	37.09	36.61	37.32

Results by age and chemical subgroup level (ATC-4) are presented in Table 2. Penicillins, including the beta-lactamase inhibitors (J01CR), made up the largest proportion of usage at about 25% and >6 DID. The proportions of antibiotic consumed showed similar trends from 2007 to 2014. Extended-spectrum penicillins (J01CA), combinations of penicillins including beta-lactamase inhibitors (J01CR), third-generation cephalosporins (J01DD), and macrolides (J01FA) all showed a noticeably higher level of usage in children ≤6 years old. In particular, DID values for combinations of penicillins, including beta-lactamase inhibitors (J01CR) and third-generation cephalosporins (J01DD), increased every year (Table 2).

**Table 2:** Total antibiotic consumption by ATC code and age group (daily defined dose/1,000 inhabitants/day).

ATC 4-level (chemical subgroup level)	2007	2008	2009	2010	2011	2012	2013	2014
**Penicillins with extended spectrum (J01CA)**								
**≤ 6 years old**	7.15	6.85	7.15	8.40	8.78	10.05	10.61	10.79
**07–19 years old**	2.69	2.38	2.46	2.42	2.23	2.29	2.30	2.48
**20–64 years old**	3.58	3.25	3.04	3.01	2.94	2.92	2.83	2.82
**≥ 65 years old**	5.05	4.70	4.42	4.39	4.28	4.33	4.31	4.32
**Total**	3.82	3.49	3.36	3.41	3.35	3.45	3.43	3.47
**Combinations of penicillins (J01CR)**								
**≤ 6 years old**	21.84	23.91	24.23	27.45	26.35	28.28	28.42	27.79
**07–19 years old**	5.74	6.50	7.55	7.48	6.64	6.68	6.82	7.55
**20–64 years old**	3.54	4.27	4.46	4.55	4.37	4.47	4.50	4.88
**≥ 65 years old**	4.15	5.01	5.17	5.23	5.03	5.28	5.32	5.68
**Total**	5.28	6.06	6.36	6.60	6.23	6.44	6.49	6.83
**First & second-generation cephalosporins (J01DC)**								
**≤ 6 years old**	7.12	7.63	7.91	8.12	6.67	6.52	6.03	5.56
**07–19 years old**	4.33	4.82	5.77	6.05	5.42	5.63	5.67	6.02
**20–64 years old**	5.11	5.46	5.74	5.97	5.81	6.04	6.14	6.40
**≥ 65 years old**	8.08	8.64	8.84	8.99	8.63	9.04	9.12	9.36
**Total**	5.41	5.82	6.22	6.46	6.12	6.36	6.43	6.66
**Third & fourth-generation cephalosporins (J01DD)**								
**≤ 6 years old**	2.67	3.06	3.49	4.50	4.62	5.26	5.26	5.33
**07–19 years old**	0.56	0.70	0.90	0.94	0.98	1.03	1.08	1.24
**20–64 years old**	0.84	0.90	0.95	0.99	1.02	1.08	1.07	1.12
**≥ 65 years old**	2.69	2.89	3.00	3.13	3.20	3.42	3.32	3.40
**Total**	1.10	1.22	1.33	1.45	1.50	1.61	1.62	1.69
**Macrolides (J01FA)**								
**≤ 6 years old**	7.73	7.97	8.79	10.54	11.99	10.38	9.13	9.51
**07–19 years old**	2.23	2.58	3.39	3.78	4.03	3.76	3.71	4.44
**20–64 years old**	1.99	2.27	2.60	2.80	2.86	3.03	3.12	3.45
**≥ 65 years old**	3.52	3.92	4.11	4.30	4.17	4.47	4.38	4.72
**Total**	2.59	2.88	3.30	3.63	3.79	3.79	3.75	4.14
**Fluoroquinolones (J01MA)**								
**≤ 6 years old**	0.06	0.05	0.04	0.00	0.00	0.00	0.00	0.01
**07–19 years old**	0.68	0.66	0.66	0.21	0.19	0.20	0.17	0.13
**20–64 years old**	2.89	2.91	2.86	2.80	2.75	2.82	2.67	2.50
**≥ 65 years old**	6.09	6.42	6.41	6.37	6.31	6.66	6.46	6.20
**Total**	2.62	2.69	2.68	2.58	2.56	2.68	2.59	2.47
**Others**								
**≤ 6 years old**	0.44	0.39	0.35	0.36	0.31	0.28	0.24	0.22
**07–19 years old**	1.28	1.24	1.26	1.28	1.28	1.30	1.28	1.29
**20–64 years old**	3.01	2.94	2.81	2.80	2.73	2.71	2.61	2.63
**≥ 65 years old**	4.36	4.32	4.13	4.08	3.83	3.90	3.70	3.66
**Total**	2.66	2.62	2.52	2.53	2.46	2.47	2.39	2.41

Note: The number of inhabitants were based on the number of each age group

DID, DDD/1,000 inhabitants/day where DDD represents WHO defined daily doses

### Monthly antibiotic usage trends

**Fig 1:**
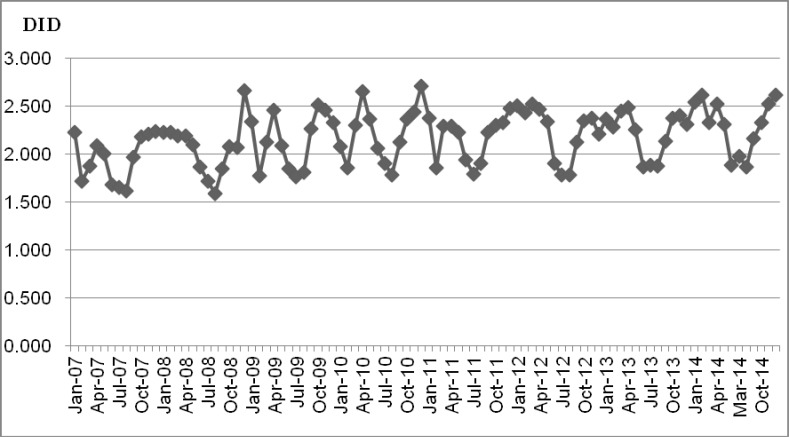
Seasonal variation in antibiotic consumption. DID, daily defined dose/1,000 inhabitants/day, based on WHO-defined daily doses

The year-on year difference were slight, but in overall, antibiotic usage was highest in April, October, and November. In 2013, usage showed troughs in June (1.871 DID), July (1.882 DID), and August (1.872 DID), and was highest in December (2.309 DID), January (2.367 DID), and February (2.283 DID) (Fig 1 and S1 Table).

**Fig 2:**
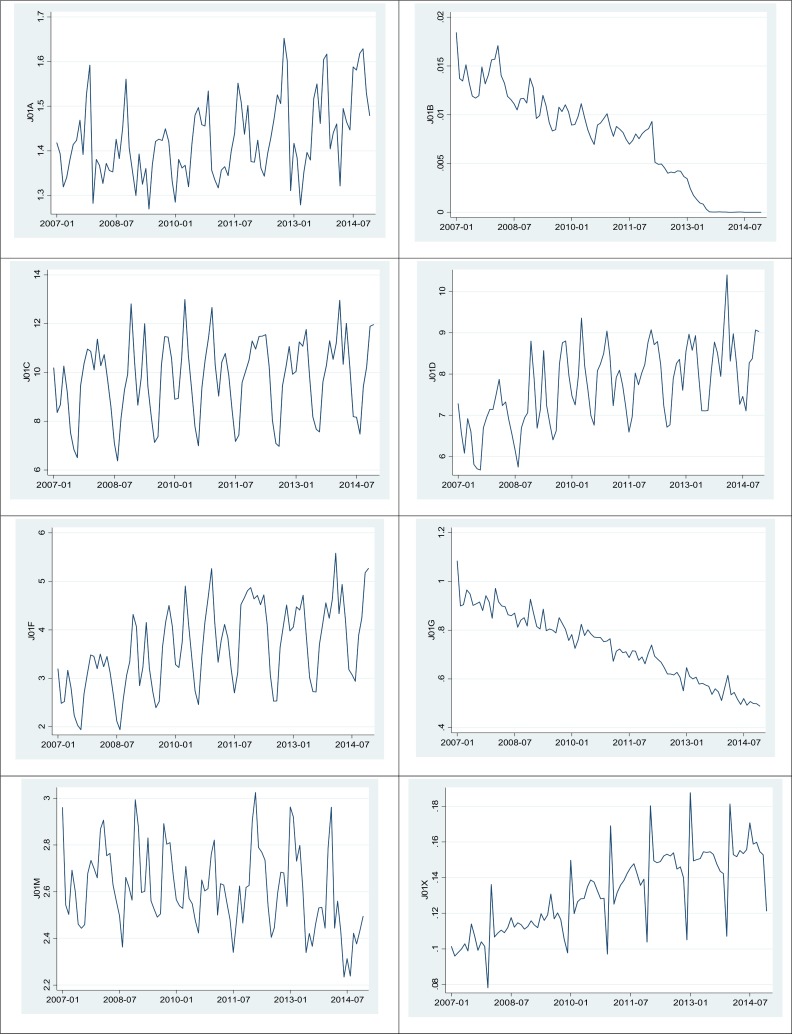
Antibiotic use from 2007 to 2014 based on the third level of ATC (the unit of Y is DID/month, where DID is the daily defined dose/1,000 inhabitants/day, based on WHO-defined daily doses). J01A: tetracyclines, J01B: amphenicols, J01C: beta-lactam antibacterials, penicillins, J01D: other beta-lactam antibacterials, J01F: macrolides, lincosamides and streptogramins, J01G: aminoglycoside antibacterials, J01M: quinolone antibacterials, J01X: other antibacterials

Fig 2 shows the time trend of antibiotic usage based on the pharmacological subgroup level (ATC-3). amphenicols (J01B) and aminoglycoside antibacterials (J01G) showed a noticeable reduction in usage. The DIDs of beta-lactam antibacterials, penicillins (J01C), other beta-lactam antibacterials (J01D), lincosamides and streptogramins (J01F), quinolone antibacterials (J01M), and other antibacterials (J01X) increased over time.

Table 3 shows the estimate of time trend by month and ATC-3 level from the ARMA model. The autoregressive and moving average of order one was applied in all models, but first differencing was used only for J01B (amphenicols) and J01G (aminoglycoside antibacterials) subgroups based on the Dickey-Fuller test results. There was positive correlation in the use of antibiotics use from one month to the next for all pharmacological subgroups except for J01G (aminoglycoside antibacterial) and J01M (quinolone antibacterials) subgroups, and the positive associations were all strong enough to warrant rejection of status quo of each antibiotic use at the 5% level (Table 3).

**Table 3:** Estimation results for the autoregressive moving average model for antibiotic use (defined daily doses per 1,000 inhabitants per day) ATC-3 level.

ARMA	J01A	J01B	J01C	J01D	J01E	J01F	J01G	J01M	J01X
**L.ar (Std)**	0.6017^***^	0.7149^***^	0.3659^**^	0.5203^***^	0.7053^***^	0.5554^***^	0.0486	0.0766	0.9933^***^
	(0.1755)	(0.0971)	(0.1549)	(0.1798)	(0.0882)	(0.1652)	(0.1376)	(0.6008)	(0.0059)
**L.ma (Std)**	-0.0959	-1.0000^***^	0.4613^***^	0.3401	-1.7054^***^	0.5019^**^	-1.0000^***^	0.5991	-0.8352^***^
	(0.1819)	(0.0001)	(0.1577)	(0.2462)	(0.5484)	(0.2487)	(0.0000)	(0.6300)	(0.0267)
**Observations**	96	94	96	96	72	96	96	96	96
**p-value from Dickey-Fuller test for unit root process**	0.0000	0.4514	0.0001	0.0017	0.0000	0.0106	0.2326	0.0000	0.0000
**p**	1	1	1	1	1	1	1	1	1
**d**	0	1	0	0	0	0	1	0	0
**q**	1	1	1	1	1	1	1	1	1

Note:

a. J01A: tetracyclines, J01B: amphenicols, J01C: beta-lactam antibacterials, penicillins, J01D: other beta-lactam antibacterials, J01F: macrolides, lincosamides and streptogramins, J01G: aminoglycoside antibacterials, J01M: quinolone antibacterials, J01X: other antibacterials

b. Std: standard errors in parentheses

c. * p<0.1, ** p<0.05, *** p<0.0001

d. The null hypothesis of the Dickey-Fuller test is a unit root process in the y (antibiotic use).

e. p and q represent the order of the autoregressive process and moving average process, respectively, and d represents the order of differencing

## Discussion

The aim of the present study was to investigate the epidemiological characteristics of antibiotic use at a national level using representative claims data of the entire population of South Korea from 2007 to 2014. In South Korea, antibiotics usage alone is far higher than in other OECD countries (average, 20.6 DID in 2012) and Europe (average, 19.1 DID in 2012) [4]. Despite the high utilization of antibiotics, the utilization of other medications in South Korea is relatively low compared to other OECD countries. The analysis can be performed at several levels within the ATC classification. In this paper, the analysis was conducted at the pharmacological subgroup (ATC-3) level or the chemical subgroup (ATC-4) level.

The findings of the current study show a relatively high use of penicillin-class antibiotics in the outpatient setting, and a high use of aminoglycoside- and cephalosporin-class antibiotics in the inpatient setting. Women showed a slightly higher total antibiotic use than did men. In terms of age, antibiotics were most frequently used in children ≤6 years old, followed by patients ≥65 years old. Antibiotic usage was least frequent in the 7–19 year old age group. The most frequently prescribed antibiotics in the ≤6 years old age group, based on data from 2014, were combinations of penicillins, including beta-lactamase inhibitors (J01CR). In 2013, Sohn et al. reported that antibiotic use was higher in infants and children than in the elderly [14]. The results of the present study are consistent with the results of previous studies in South Korea. Conversely, in Sweden, antibiotic use is lower in children compared to other age groups and is decreasing annually [15]; this is a very different pattern from that seen in South Korea.

With regard to the choice of antibiotics prescribed, broad-spectrum antibiotics were prescribed more frequently than other antibiotics usually recommended as first-line therapy. There is a large difference between the annual patterns of South Korean antibiotic usage and resistance for the individual ATC antibiotic classes when compared with those of Sweden [15]. For example, in Sweden, beta-lactamase sensitive penicillins (J01CE) accounted for 29.7% of antibiotic use (3.49 DID), while extended-spectrum penicillins (J01CA) and combinations of penicillins, including beta-lactamase inhibitors (J01CR), constituted 11% (1.31 DID) and 2% (0.23 DID) of usage, respectively [15]. However, in Korea, beta-lactamase sensitive penicillins (J01CE) accounted for 0% of usage, while extended-spectrum penicillins (J01CA) and combinations of penicillins, including beta-lactamase inhibitors (J01CR), accounted for 12.9% and 24.3% of usage, respectively. While second through fourth generation cephalosporins (J01DB-E) accounted for 30% (8.01 DID) of antibiotic use in Korea, they accounted for only 1.4% of use (0.16 DID) in Sweden.

The high utilization of antibiotics in children ≤6 years old (59.21 DID) in South Korea is a significant problem. In contrast, antibiotic usage was 23.15 DID for 7–19 year olds and 37.32 DID for patients ≥ 65 years old. Sweden shows a completely different pattern of antibiotic use relative to age.

The present study aimed to measure antibiotic usage as accurately as possible by using health insurance claims data, including almost all Koreans as beneficiaries. The data regarding the epidemiological characteristics of antibiotic use in the present study are particularly useful. Our findings identify the groups that most utilize antibiotics, and our results can be used as the basis for instituting control policies targeting these groups. It should be noted that there are a large number of drugs used in South Korea that have no DDD or ATC category, including traditional Korean medicines, new medications, and combination medications. These drugs were not included in the current study, and thus, estimates from the current study may underestimate the actual usage of certain drugs.

Our study has some limitations. First, since we utilized the National Health Insurance claim database, utilization information regarding over-the-counter medicines and medications not listed in the database is not available, thus we might have underestimated the DID of antibiotics. However, all antibiotics are listed in the database, and patients should have access to antibiotics without physicians’ prescribing in Korea. In addition, we were not able to compare adverse effects such as antimicrobial resistance. Further study exploring antibiotics use and resistance is needed.

In conclusion, this study provides valuable statistics regarding antibiotic usage in South Korea. Antibiotics use in the ≤6 years old age group showed the highest level of usage at 59.21 DID in 2014, and showed an increasing trend each year. DIDs of beta-lactam antibacterials, penicillins (J01C), other beta-lactam antibacterials (J01D), lincosamides and streptogramins (J01F), quinolone antibacterials (J01M), and other antibacterials (J01X) increased significantly over time. This result is important for guiding health policy with regard to antibiotic usage.

## Supporting information

S1 Table Minimal data set. (PDF)
