# Supplementary material for: Antibiotic use in South Korea from 2007 to 2014: A health insurance database-generated time series analysis
Source: PLoS One. 2017 May 17;12(5):e0177435. doi: 10.1371/journal.pone.0177435 (PMC5435228; doi:10.1371/journal.pone.0177435)
Supplement: S1 Table — (PDF) [file pone.0177435.s001.pdf]

Gender	Age group	Service type	ATC3level	DDD07	DDD08	DDD09	DDD10	DDD11	DDD12	DDD13	DDD14
1	00~06	1	J01A	34.3	16.3	27.0	17.6	394.9	36.7	21.3	42.7
1	00~06	1	J01B	17.2	4.1	2.0	11.3	3.9	7.0		
1	00~06	1	J01C	437101.3	441117.5	459960.2	564533.6	536280.5	527412.8	522916.8	523682.3
1	00~06	1	J01D	385675.1	419249.1	411742.6	501804.5	486971.7	496849.3	494135.0	491086.6
1	00~06	1	J01E	47778.5	50100.0	55814.8	63386.0	61895.4	52476.1	12288.6	8919.0
1	00~06	1	J01F	266563.7	216859.7	229694.0	299938.7	429530.6	352890.7	287622.6	343124.6
1	00~06	1	J01G	131760.4	126739.5	120589.7	131225.7	116103.3	106205.1	89963.1	81982.6
1	00~06	1	J01M	690.4	557.3	599.4	102.2	228.6	181.4	308.0	715.0
1	00~06	1	J01X	7904.5	9641.6	9402.7	9549.1	10242.5	11256.9	10375.7	10615.6
1	00~06	2	J01A	4534.7	1527.9	71.5	41.0	938.5	150.7	26.2	15.0
1	00~06	2	J01B	2684.6	1740.0	1167.6	1178.1	1136.0	198.2	10.2	
1	00~06	2	J01C	19388510.1	19760845.4	19734422.8	22111016.5	21692206.9	23759561.9	24383590.9	24009283.7
1	00~06	2	J01D	6322620.6	6658913.9	6960480.8	7539387.2	6733455.6	7046459.4	6768265.6	6478235.5
1	00~06	2	J01E	1598298.7	1284952.9	1174893.3	1233235.7	1169156.4	1094304.5	298266.6	235099.5
1	00~06	2	J01F	5064804.4	5075065.0	5486646.1	6427388.4	7208306.4	6275098.4	5576164.7	5739625.4
1	00~06	2	J01G	104274.5	83027.9	64625.5	60848.1	45469.3	39412.0	31585.3	24545.6
1	00~06	2	J01M	39249.1	32538.7	26498.2	1198.2	327.1	758.9	401.9	7490.7
1	00~06	2	J01X	4449.5	1473.4	403.3	503.4	585.7	363.9	280.8	246.2
1	07~19	1	J01A	6508.4	5592.3	6063.0	6664.4	6446.1	6519.1	6688.2	5932.9
1	07~19	1	J01B	54.4	97.4	65.8	27.1	24.9	42.8	3.5	
1	07~19	1	J01C	262764.7	290556.4	332756.6	317586.9	337463.5	293936.4	277414.8	290969.3
1	07~19	1	J01D	837766.4	919997.3	960661.9	982021.5	1021812.2	972288.6	893974.6	873686.9
1	07~19	1	J01E	38164.7	38276.1	38719.1	34548.5	36936.4	30144.0	22983.3	22888.9
1	07~19	1	J01F	118005.7	104484.7	147021.4	153022.4	268274.4	154255.0	121173.9	166664.4
1	07~19	1	J01G	211412.8	200750.6	196366.0	175768.5	167984.0	140849.5	111177.8	95470.3
1	07~19	1	J01M	45782.4	50580.9	57288.9	33853.1	33639.9	33323.6	33141.8	32548.3
1	07~19	1	J01X	37548.7	42546.6	48391.2	51827.1	55175.8	57316.4	49501.3	46045.1
1	07~19	2	J01A	1385343.2	1362909.0	1309908.3	1403065.8	1409213.4	1400954.0	1380123.2	1377313.7
1	07~19	2	J01B	16130.2	13614.9	12039.8	10777.9	7758.0	2918.7	514.0	
1	07~19	2	J01C	13942953.9	14730160.1	16333705.7	15735874.8	13700495.2	13462866.1	13203213.3	13969726.8
1	07~19	2	J01D	7580619.7	8511290.3	10139901.2	10372167.8	9137519.9	9219940.9	9020648.4	9374623.7
1	07~19	2	J01E	639097.6	561167.7	532283.7	510964.4	467348.0	429529.3	276098.6	253789.5
1	07~19	2	J01F	3850927.3	4494155.5	5708923.7	6173648.4	6218399.1	5703545.5	5481061.3	6231731.9
1	07~19	2	J01G	468767.1	442799.3	430156.2	394927.1	330547.6	293681.0	252781.5	222636.0
1	07~19	2	J01M	1062580.6	1009081.9	981901.3	262674.6	239129.1	239699.1	189447.9	139275.6
1	07~19	2	J01X	8175.0	6673.6	6859.3	7591.7	6661.3	5543.5	4449.5	3818.2
1	20~64	1	J01A	126399.9	118609.1	125824.2	131682.8	127551.2	139729.7	139436.6	133087.1
1	20~64	1	J01B	1585.5	1149.0	501.6	549.7	558.2	333.1	64.3	1.0
1	20~64	1	J01C	1254492.7	1312224.4	1327446.4	1360306.9	1417810.8	1456475.6	1458457.2	1474612.2
1	20~64	1	J01D	6196589.6	6560336.1	6636186.4	6906551.7	7150740.7	7312027.2	7284200.7	7059998.2
1	20~64	1	J01E	98132.1	109528.3	100602.5	102012.2	110987.9	113452.2	114278.9	114522.3
1	20~64	1	J01F	570054.7	585253.0	559648.6	572405.6	632714.7	601080.0	554412.0	543353.0
1	20~64	1	J01G	1647457.1	1492055.3	1340620.8	1191955.9	1063067.7	939887.8	811759.0	691560.5
1	20~64	1	J01M	1331926.2	1477972.4	1530682.9	1594772.2	1707458.4	1767629.6	1807409.2	1844864.3
1	20~64	1	J01X	492811.2	572023.0	570803.6	639791.8	688283.8	720812.2	719616.2	739423.7
1	20~64	2	J01A	10450484.6	10138896.6	9755915.0	9729246.3	9661565.2	9767574.0	9629315.7	10011412.9
1	20~64	2	J01B	64747.4	58960.4	46013.6	39678.5	36772.4	22424.9	4357.0	3.8
1	20~64	2	J01C	34814432.0	37181157.1	37349176.9	38230352.9	37921837.5	38998132.8	39254534.0	41621530.7
1	20~64	2	J01D	23640373.6	25177396.8	26526950.5	27941104.1	27792016.3	29569085.2	30803762.6	32671090.3
1	20~64	2	J01E	875813.6	803267.1	727836.0	674113.4	651132.7	658129.8	625362.7	619868.7
1	20~64	2	J01F	10751458.3	11966980.2	13121404.9	14273373.1	14650986.6	15612695.8	16137249.1	17839709.8
1	20~64	2	J01G	3082678.0	2978914.7	2760560.8	2699216.7	2526170.7	2394693.9	2199579.5	2029105.8
1	20~64	2	J01M	14891709.0	14947223.4	14641191.1	14235024.5	14039578.0	14417342.5	13720602.1	12776336.1
1	20~64	2	J01X	80671.5	70528.4	68478.4	68728.4	64625.8	52740.2	50913.4	53237.6
1	>=65	1	J01A	74405.2	74029.4	83373.7	82009.6	78433.2	93704.8	98779.3	93430.8
1	>=65	1	J01B	1585.7	755.5	522.3	587.7	469.3	595.1	84.9	
1	>=65	1	J01C	663387.7	710283.3	711538.4	768980.8	845147.3	955053.8	1055581.0	1161600.4
1	>=65	1	J01D	3030597.9	3322071.8	3396550.3	3639659.4	3799247.2	4119186.9	4187974.5	4315230.5
1	>=65	1	J01E	55163.6	53477.1	55348.1	58814.6	62970.0	75405.8	71332.6	79996.3
1	>=65	1	J01F	503374.3	557784.9	549385.8	553376.1	570399.6	600061.8	557575.5	546234.2
1	>=65	1	J01G	781746.4	707191.8	637874.2	571806.1	493067.0	463152.6	405831.3	362440.3
1	>=65	1	J01M	979518.8	1158474.4	1266375.5	1406779.1	1552777.1	1757485.8	1851827.6	1943796.3
1	>=65	1	J01X	341403.8	433560.3	439756.0	510777.5	559942.6	644800.2	677771.0	737636.8
1	>=65	2	J01A	1100803.7	1203608.0	1206382.4	1278593.8	1267632.7	1371428.5	1384717.1	1485420.4
1	>=65	2	J01B	22293.1	23901.5	21076.1	19994.8	18497.5	15299.0	2488.8	8.5
1	>=65	2	J01C	6500622.9	7287273.8	7547950.4	7943292.5	7999107.1	8633389.3	9065886.6	9814943.6
1	>=65	2	J01D	5326788.5	6140917.0	6677514.9	7163178.7	7277504.1	8095115.0	8601793.2	9329727.2
1	>=65	2	J01E	195270.5	196390.0	201301.2	203624.3	203295.8	209084.9	215052.9	233112.8
1	>=65	2	J01F	2818091.0	3254766.3	3560686.5	3916181.2	3861853.8	4223061.0	4456083.5	4999075.4
1	>=65	2	J01G	661539.4	714145.9	708645.0	706495.8	662021.6	655179.7	606246.5	577622.2

Gender	Age group	Service type	ATC3level	DDD07	DDD08	DDD09	DDD10	DDD11	DDD12	DDD13	DDD14
1	>=65	2	J01M	3775261.6	4262693.9	4390969.9	4443425.3	4494038.3	4897347.2	4900326.5	4818367.4
1	>=65	2	J01X	14942.5	13179.4	12889.2	16906.6	18185.3	17073.5	16999.2	22586.1
2	00~06	1	J01A	82.3	55.0	21.1	34.2	211.9	23.7	28.2	19.3
2	00~06	1	J01B	15.0	2.7	4.3	3.3	3.8	0.1		
2	00~06	1	J01C	333720.4	335489.8	351353.8	448696.2	436121.1	425222.3	423843.7	438350.8
2	00~06	1	J01D	284620.9	305968.1	308574.7	387657.0	389168.7	387594.8	391332.0	409749.8
2	00~06	1	J01E	39936.0	38747.2	44788.1	50127.2	44653.1	41663.8	10454.8	6838.9
2	00~06	1	J01F	220066.4	179527.6	185790.8	252920.1	372183.8	307760.8	253585.3	310148.0
2	00~06	1	J01G	103512.4	99791.0	96083.7	107014.8	95247.9	87105.0	75044.7	69921.0
2	00~06	1	J01M	457.5	391.3	252.9	137.2	138.5	93.1	255.7	216.1
2	00~06	1	J01X	6099.9	6313.7	6387.7	6365.1	8234.0	7665.4	7856.6	8395.6
2	00~06	2	J01A	2868.2	132.5	65.7	182.2	1154.0	38.0	40.0	28.5
2	00~06	2	J01B	2015.9	1415.1	952.9	963.1	824.7	151.3	11.8	
2	00~06	2	J01C	16030864.5	16572486.2	16531616.5	18772052.5	18402270.7	20228111.9	20691010.0	20451511.8
2	00~06	2	J01D	5233216.7	5513548.5	5789500.2	6329570.0	5606235.8	5888583.6	5668558.7	5454336.3
2	00~06	2	J01E	1312097.7	1052441.7	963458.9	996161.4	954894.7	911679.9	244018.6	187548.1
2	00~06	2	J01F	4173910.7	4208918.4	4542578.4	5391827.3	6040009.5	5275032.1	4676776.9	4840355.8
2	00~06	2	J01G	79328.4	60874.3	47165.7	44562.6	32183.0	28735.2	22681.3	17501.1
2	00~06	2	J01M	28919.8	26051.4	20178.1	1083.8	190.4	445.2	300.5	195.3
2	00~06	2	J01X	3053.4	1083.0	263.6	446.6	485.6	300.0	399.0	238.7
2	07~19	1	J01A	8244.2	9290.8	12275.5	11532.9	15464.3	14223.0	14975.4	16090.2
2	07~19	1	J01B	107.8	60.7	45.9	42.3	66.1	43.2	3.3	
2	07~19	1	J01C	187747.3	208879.4	239743.9	242375.6	270620.5	228107.5	216398.6	234313.8
2	07~19	1	J01D	434653.2	485116.0	521026.0	546460.3	594723.6	552334.9	512187.7	523751.0
2	07~19	1	J01E	23151.7	27520.4	28882.4	29094.0	26240.0	23377.0	15530.5	17042.6
2	07~19	1	J01F	95182.0	79200.3	108352.5	132198.5	255161.5	138666.6	106745.8	160756.9
2	07~19	1	J01G	122314.1	118412.2	122866.3	111786.1	108401.7	92053.3	75137.4	67549.2
2	07~19	1	J01M	37094.0	40527.0	44430.5	27350.7	29433.9	28426.4	26702.4	28152.3
2	07~19	1	J01X	26238.4	31593.5	32976.1	34702.6	39298.3	40481.2	35324.8	35603.5
2	07~19	2	J01A	1044403.7	1007191.9	1044811.9	1077436.5	1124571.2	1183410.5	1172719.5	1176403.9
2	07~19	2	J01B	13945.2	11193.1	9687.6	8946.6	6349.4	2408.2	431.5	0.1
2	07~19	2	J01C	11879662.9	12351058.6	13841029.3	13478372.2	11723207.8	11476649.8	11224206.2	12003001.3
2	07~19	2	J01D	6411705.9	7252232.7	8874893.2	9153820.5	8060732.4	8175012.2	8047315.9	8434636.1
2	07~19	2	J01E	560251.0	477313.1	454833.0	439400.8	396360.8	363331.0	226450.2	203369.0
2	07~19	2	J01F	3144500.4	3603175.6	4698574.0	5138790.3	5255799.6	4837123.5	4601260.5	5325048.3
2	07~19	2	J01G	344485.7	328859.0	323215.2	295551.4	247563.5	223943.0	194902.8	172616.1
2	07~19	2	J01M	981096.1	948855.6	947489.5	296429.1	268293.9	262859.1	212604.1	155349.8
2	07~19	2	J01X	4644.1	3151.6	2861.6	3868.2	3503.0	2914.1	2925.4	2573.7
2	20~64	1	J01A	199203.0	195773.3	210853.4	214665.0	217148.4	244787.0	250934.9	229221.2
2	20~64	1	J01B	3447.5	2146.5	1042.0	908.8	882.7	424.6	103.5	
2	20~64	1	J01C	2072818.2	1990985.1	1900466.7	1969880.2	1937439.0	1878510.8	1680659.5	1646984.9
2	20~64	1	J01D	6622716.4	6937030.4	7062292.8	7493609.7	7799185.7	7787420.3	7292033.2	6815365.3
2	20~64	1	J01E	67697.5	69837.2	66206.3	65630.0	69135.4	74097.0	73490.2	74065.4
2	20~64	1	J01F	407863.2	426963.5	427238.8	468615.3	565770.6	534528.3	480412.8	510771.6
2	20~64	1	J01G	1951990.6	1767709.1	1630775.3	1516187.9	1388261.1	1168808.1	913989.8	785465.2
2	20~64	1	J01M	1189435.5	1301675.2	1355798.4	1425353.2	1523379.7	1606019.1	1618984.3	1627665.2
2	20~64	1	J01X	384244.0	436720.6	437598.9	475568.5	503350.8	529485.0	507023.4	505104.5
2	20~64	2	J01A	9377309.6	9581760.4	9596236.7	10110947.7	10417883.0	10915450.7	11047912.9	11673193.9
2	20~64	2	J01B	83913.5	77651.8	60869.3	54882.5	46497.4	28282.6	5311.3	6.0
2	20~64	2	J01C	44621946.5	47569409.8	47816054.0	48179413.5	46465445.1	47076876.1	46919168.3	49743365.0
2	20~64	2	J01D	32876244.1	36131152.4	38925730.1	40654171.1	39732908.2	41876259.0	43018218.6	46126559.1
2	20~64	2	J01E	1199463.0	1109430.4	1008547.1	993281.2	914486.1	903411.3	881417.4	907052.9
2	20~64	2	J01F	13324002.7	15508892.5	18278909.9	19621703.4	20080270.2	21509083.4	22281999.9	24905626.0
2	20~64	2	J01G	4729269.0	4659281.1	4331144.4	4182787.9	3906655.9	3662058.1	3377264.4	3108320.5
2	20~64	2	J01M	16115621.9	16354972.6	16207594.6	16010371.8	15702941.0	16262689.5	15413913.5	14391138.3
2	20~64	2	J01X	48829.6	45527.2	47853.5	54529.1	60107.0	63334.8	64907.8	71616.8
2	>=65	1	J01A	137862.5	117192.0	118767.9	113907.0	108967.6	123145.8	130592.6	122962.8
2	>=65	1	J01B	3095.6	1744.9	803.6	821.2	826.2	815.6	140.8	6.3
2	>=65	1	J01C	770502.8	710565.9	679965.2	730305.2	767470.6	879677.1	929985.9	999384.5
2	>=65	1	J01D	3834205.9	3949036.3	4067225.3	4360813.4	4503036.8	4943893.2	5049004.2	5087981.6
2	>=65	1	J01E	93599.0	55882.8	55377.9	53095.6	59250.5	66086.2	64379.2	68719.9
2	>=65	1	J01F	421103.5	441649.3	435066.6	426811.1	458951.7	614245.4	460746.4	459110.9
2	>=65	1	J01G	986807.8	832673.3	756367.3	677394.7	580835.1	554517.8	482685.6	424458.9
2	>=65	1	J01M	1290027.1	1273875.4	1355100.8	1483415.8	1607235.5	1816513.2	1879284.4	1962783.0
2	>=65	1	J01X	298396.5	350301.2	370855.8	429892.9	471688.6	532598.3	569180.7	600495.3
2	>=65	2	J01A	1133320.3	1166740.8	1189526.2	1251893.9	1204436.2	1267067.5	1272527.2	1342134.4
2	>=65	2	J01B	31790.3	34352.4	30189.6	25052.5	24530.1	19753.6	3192.5	2.8
2	>=65	2	J01C	8286215.6	9205462.2	9477953.8	9710148.4	9611646.3	10204613.1	10543978.6	11337413.3
2	>=65	2	J01D	7092963.6	8229545.4	9020586.1	9506490.3	9483034.6	10459595.8	10976171.7	12089566.8
2	>=65	2	J01E	346291.1	340806.6	337782.4	340310.6	322545.7	331289.4	338916.8	366269.6

Gender	Age group	Service type	ATC3level	DDD07	DDD08	DDD09	DDD10	DDD11	DDD12	DDD13	DDD14
2	>=65	2	J01F	3195484.0	3740546.4	4099737.7	4431343.7	4447973.2	4922068.3	5070185.5	5734270.5
2	>=65	2	J01G	1026146.6	1101168.9	1092954.4	1079423.9	1016636.2	997443.6	919484.8	871143.6
2	>=65	2	J01M	4696216.7	5147175.1	5278274.1	5348481.9	5382537.6	5844663.8	5834978.3	5725303.5
2	>=65	2	J01X	15243.5	14206.6	14224.7	18773.9	19448.9	19747.7	24184.1	27142.7
